# Clinical changes induced by allergen immunotherapy with dermatophagoides pteronyssinus in local allergic rhinitis

**DOI:** 10.1186/2045-7022-5-S4-O4

**Published:** 2015-06-26

**Authors:** Carmen Rondon, Paloma Campo, Maria Jose Torres, Luisa Galindo, Natalia Blanca-Lopez, Gabriela Canto, Cristobalina Mayorga, Miguel Blanca

**Affiliations:** 1 Regional University Hospital of Malaga, IBIMA, UMA, Allergy Unit, Malaga, Spain; 2 Regional University Hospital of Malaga, IBIMA, UMA, Research Laboratory, Malaga, Spain; 3 Infanta Leonor Hospital, Allergy Service, Madrid, Spain

## Background

Allergen immunotherapy (AIT) is the etiologic treatment of the allergic respiratory disease and has the ability to modify their natural course. In this study we investigated the efficacy and safety of subcutaneous AIT with Dermatophagoides pteronyssinus (DP) in local allergic rhinitis (LAR).

## Methods

Thirty-six subjects with LAR to DP were selected to participate in a double-blind, placebo-controlled, parallel-group, phase II clinical trial of subcutaneous AIT in LAR. The patients were randomized to receive AIT-DP with Pangramin PLUS, ALK, DP, or placebo for a period of 24 months. The primary endpoint was total symptoms (TSS) and total medication scores (TMS). Secondary endpoints were: total combined symptom+medication scores (TCS), daily symptoms score (DSS), daily medication score (DMS), medication free days (MFD), skin testing, nasal allergen provocation test (NAPT-DP), and adverse events. Serum and nasal lavage samples were obtained for immunological studies.

## Results

AIT-DP induced a clinically relevant and significant improvement compared to placebo with a 47% of reduction in TSS (0.60 vs 1.14; p<0.001) and a 51.2% in TMS (0.65 vs 1.34; p=0.002). At 6-12-18-24 months significant improvements in TCS (p=0.046; p=0.037; p=0.011; p=0.007) and DSS (p=0.003; p=0.012; p<0.001; p<0.001); and at 24 months in DMS (p=0.014), and MFD (p=0.031) compared to placebo were also observed. AIT-DP induced an increase in nasal tolerance to NPAT-DP at 6-12-18-24 months (p=0.003; p<0.001; p<0.001; p<0.001) compared to placebo, with negative NAPT-DP in the 50% of patients. One patient with AIT-DP had a local moderate reaction solved without systemic treatment, no systemic reactions occurred.

## Conclusion

Subcutaneous allergen immunotherapy with Dermatophagoides Pteronyssinus has demonstrated to be an effective and well-tolerated treatment in LAR. This phase II study provides the indication for AIT in LAR. To our knowledge this is the first study carried out in patients with LAR in patients sensitised to house dust mite.

